# Vitamin B12 prescribing from 2015 to 2024 in English general practice: an observational study to investigate the switch from injections to tablets

**DOI:** 10.1136/bmjopen-2024-093748

**Published:** 2025-02-12

**Authors:** Nikita Tank, Christopher Wood, Anand Ahankari, Brian Mackenna, Alex Walker, Agnieszka Lemanska

**Affiliations:** 1 Cardiff University, Cardiff, UK; 2 University of Oxford, Oxford, UK; 3 Manchester Metropolitan University, Manchester, UK; 4 NHS England, Redditch, UK; 5 University of Surrey, Guildford, UK

**Keywords:** COVID-19, Primary Care, Health policy, Prescriptions, Drug Therapy, Retrospective Studies

## Abstract

**Abstract:**

**Background:**

Traditionally, intramuscular vitamin B_12_ injections were considered by patients and clinicians the most effective treatment option for B_12_ deficiency. The improving understanding of the condition paired with the restricted National Health Service (NHS) resources, resulted in a shift from injections towards tablets. The COVID-19 pandemic accelerated this change, while healthcare services were adapted to reduce COVID-19 transmission. This included new guidelines on vitamin B_12_ prescribing where injections were substituted by tablets.

**Objective:**

We investigated changes between 2015 and 2024 in prescribing B_12_ injections and tablets including the effect of the COVID-19 pandemic.

**Design, population and setting:**

This was an observational study of general practice in England covering 100% of the population.

**Methods:**

We used prescribing data published by the NHS Business Service Authority. Monthly prescription counts and rates per 100 000 patients were visualised in longitudinal plots from 1 January 2015 to 30 September 2024. We stratified the analysis by regions in England. Changes in yearly counts and rates were summarised using descriptive statistics. Linear regression and data from before the COVID-19 pandemic were used to model trends from 2020 to 2024 as if the pandemic had not occurred. The predicted values and their 95% CI were used to assess the effect of the pandemic.

**Results:**

The number of prescriptions for B_12_ formulations doubled in the last 10 years from 2.5 million to 5 million per year. The prescriptions for tablets increased from half a million in 2015 to 2 million in 2024. While the prescriptions for injections increased from 2 million to 3 million. In 2020, there was a sharp drop in prescriptions for injections and a simultaneous rapid increase in prescriptions for tablets coinciding with the onset of the pandemic. There were 806 031 (27%) less than expected prescriptions for injections (2 171 924 observed vs 2 977 956 predicted, 95% CI 2 905 348 to 3 050 565) and 299 834 (27%) more prescriptions for tablets (1 415 315 observed vs 1 115 481 predicted, 95% CI 1 094 350 to 1 136 612). After the 2020 drop, by 2024, injections returned to the prepandemic levels of 3 million prescriptions per year and tablets doubled from 1 million in 2019 to 2 million prescriptions in 2024.

**Conclusions:**

In this study, we document important changes to vitamin B_12_ prescribing in England over the last 10 years. Before the pandemic, injections were the medication of choice for B_12_ deficiency but there had been an ongoing debate about the benefits and cost of injections over tablets. The pandemic accelerated the switch from injections to tablets. However, these changes in the pandemic were driven by the availability of resources and not necessarily clinical evidence or patient preference. To establish best practices, more evidence is needed comparing the safety and effectiveness of injections and tablets specific to the condition being treated.

**STRENGTHS AND LIMITATIONS OF THIS STUDY**

- This is the first national study documenting patterns in the prescribing of vitamin B_12_ and focusing on a switch from injections towards tablets.
- We used a data set with 100% coverage of general practice in England and studies from other countries are needed to understand the global perspective.
- Data were current, with a wide 10-year window from 2015 to 2024, demonstrating the immediate and long-term effects of the COVID-19 pandemic.
- We used a transparent and open research approach, with data refreshed monthly facilitating future updates of this study to monitor further changes in prescribing as we continue learning about healthcare systems.
- We captured prescribing but could not capture B_12_ supplements purchased by patients online or over the counter and future studies are needed to investigate this.

## Introduction

 Vitamin B_12_ (cobalamin) is an essential component responsible for the functioning of the nervous system and the formation of red blood cells.^1 2^ There are two main factors that can lead to vitamin B_12_ deficiency, either poor dietary intake or malabsorption.^3 4^ In the UK and USA, the prevalence of B_12_ deficiency is around 6% in people below 60 years of age and estimated at 20% in people 60 and over.^5^ In most people, the condition is hidden and difficult to diagnose but affects the quality of life. In chronic and severe cases, B_12_ deficiency can lead to serious and potentially life-threatening disorders including cognitive changes, neurological damage and haematological abnormalities. This includes neuropathy, ataxia and megaloblastic anaemia (abnormally large and immature red blood cells).^6^,^8^ Pernicious anaemia is a rare but irreversible condition caused by malabsorption which can be linked to gastrointestinal autoimmune disorders or surgical interventions. It requires lifelong B_12_ supplementation and in the UK, the National Institute for Health and Care Excellence (NICE), the British National Formulary (BNF), and the National Health Service (NHS) recommend intramuscular injections of hydroxocobalamin.^4 9 10^

Traditionally, intramuscular injections were considered the most effective treatment for B_12_ deficiency which cannot be corrected with diet.^3^ However, the evidence for the effectiveness of B_12_ injections over oral treatments is limited.^11^,^15^ Two Cochrane reviews of randomised controlled trials (RCTs) found that intramuscular and oral formulations (of at least 1000 μg a day) were equally effective and safe in restoring B_12_ serum levels.^12 13^ The British Committee for Standards in Haematology guideline (2014)^14^ and the NICE (2024) guidelines on diagnosis and management of vitamin B_12_ deficiency^15^ state that oral therapy can be as suitable as injections, but this depends on the causes of the deficiency, for example, whether it was linked to dietary deficiency or to a physiological disorder.

Clinical judgement and a patient-centred approach are needed in the management of B_12_ deficiency. All resources highlight the limited quantity (a small number of RCTs) and issues around quality (risk of bias) in available evidence.^14 15^ They also concluded that the research needed to focus on assessing the effectiveness of the treatment in improving patients’ symptoms and quality of life and not only on restoring B_12_ serum levels. All guidelines reinforced the importance of appropriate dosing and patient compliance in the effectiveness of B_12_ supplementation. Oral formulations have the benefit of easier administration. However, intramuscular administration should be considered in people with severe malabsorption manifesting with haematological and neurological symptoms where an immediate restoration of serum B_12_ levels was needed.

The COVID-19 pandemic caused major disruptions and rapid changes in primary care services with an increase in remote consultations.^16^ UK healthcare providers issued interim guidelines which advocated postponing or replacing injections of vitamin B_12_ with tablets to minimise in-person appointments and reduce COVID-19 transmission.^17 18^ In this study, we assess the impact of these guidelines on prescribing in England. We used a national data set covering all general practice (GP) practices in England. The objectives were to examine prescribing patterns over time from 2015 to 2024 and to assess the immediate and long-term effects of the COVID-19 pandemic on B_12_ prescriptions and healthcare service delivery.

## Methods

### Data source

Data were extracted from the monthly prescribing information published by the NHS Business Service Authority, under the Open Government Licence.

### Study design and setting

This was a retrospective, observational study set in England. The data were from all NHS GP practices in England between 1 January 2015 and 30 September 2024.

### Outcome measures

We included the two main forms of vitamin B_12_, hydroxocobalamin (BNF code 0901020N0)^19^ and cyanocobalamin (BNF code 0901020D0).^20^ Only those two forms were available via prescriptions from the NHS. Hydroxocobalamin is an injectable form.^2 10^ Cyanocobalamin comes mainly in oral formulations, although it can also come in an injectable form. We extracted prescription data separating injectables and tablets. The list of tablet formulations can be found in the online supplemental table S1 and the list of injectable formulations can be found in the online supplemental table S2.

### Statistical analysis

We presented the outcomes in terms of crude prescription counts and rates per 100 000 registered patients. We also presented the average quantity of units per prescription (ampoules or tablets) over time. The number of registered patients per year (to serve as the denominator for yearly rates) was calculated from the monthly numbers of patients by taking an average. It was 57 117 376, 57 674 187, 58 473 838, 59 194 531, 59 908 362, 60 433 996, 60 937 210, 61 771 877, 62 586 477 and 63 302 856 for years 2015–2024, respectively. Rates were used to account for changes in the population sizes over time and the differences between regions. Rates were used also because they allow national and international comparisons between published studies.

The observed monthly prescription counts, rates and units per prescription were visualised in longitudinal plots from January 2015 to September 2024. We stratified the analysis by seven regions in England: Midlands, North East and Yorkshire, North West, South East, South West, East of England and London.^21^ To estimate the changes in prescribing over time and the effect of the COVID-19 pandemic, we compared prepandemic data from the years 2015 to 2019 to the years from 2020 to 2024. We used linear regression to model the trends between 2015 and 2019 and to predict the expected trends between 2020 and 2024. The smoothed trends in the regional prescribing were calculated using the 3-point moving average method. Yearly counts and rates were summarised using descriptive statistics. For 2024, we held data ending on 30 September 2024. Therefore, to enable comparisons of 2024 with previous years, we estimated the data for this year pro-rata by dividing the total count by 9 and multiplying it by 12.

### Software and reproducibility

To process data and undertake statistical analysis, we used R: A Language and Environment for Statistical Computing, Core Team V.4.3.2 (2023), R Foundation for Statistical Computing, Vienna, Austria (https://www.R-project.org/). The function plot_time_series in the package timetk was used for graphics. We made the data set available for download from an Open Science Framework repository.^22^

### Patient and public involvement

No formal patient and public involvement activities were undertaken as part of this study.

## Results

### National trends

**Figure 1:**
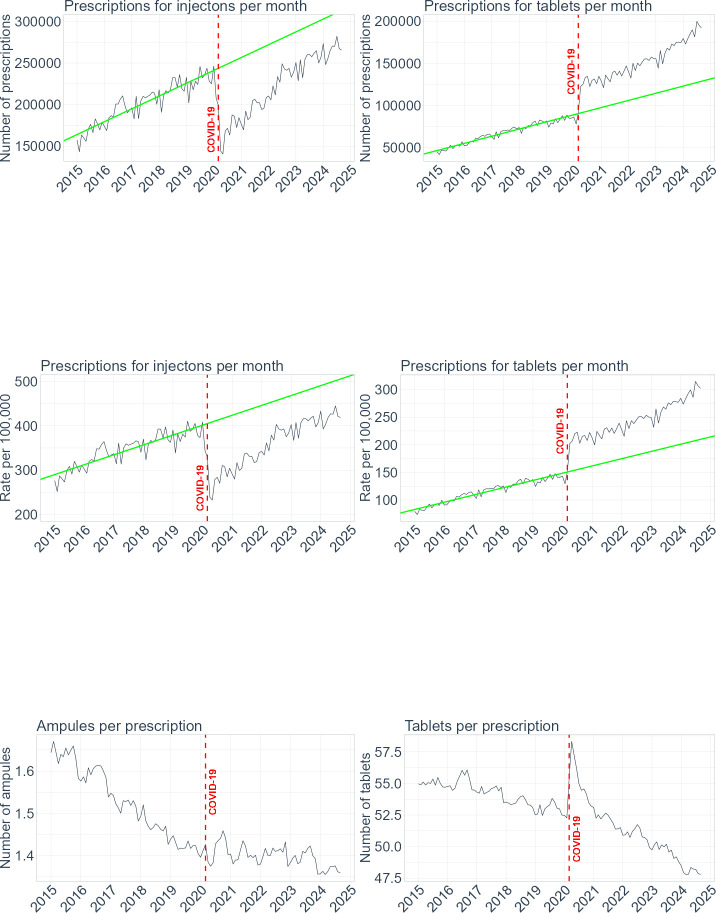
National trends in prescribing of injections (left) and tablets (right) from January 2015 to September 2024. The top two graphs show crude monthly counts of prescriptions, the middle two graphs show monthly rates per 100 000 registered patients and the bottom two graphs show the average quantities of units (ampoules and tablets) per prescription. The green solid line represents a linear model for data from January 2015 to December 2019.

We observed rapid changes in counts and rates of prescriptions starting in early 2020 for both injections and tablets (figure 1). In 2020, there were 571 393 (21%) fewer prescriptions for injections than in 2019 (table 1). Using modelled data (taking into account the longitudinal trends), we showed that in 2020 there were 806 031 (27%) fewer prescriptions for injections than there would have been if the pandemic had not occurred. The difference was statistically significant, the observed count in 2020 was 2 171 924 and the predicted was 2 977 956 (95% CI: 2 905 348 to 3 050 565). Although the deficit between observed and predicted prescriptions for injections was getting smaller over time, it remained significant until the end of the study period. In 2024, the difference was 499 845 (13%), 3 194 952 observed and 3 694 797 predicted (95% CI: 3 541 711 to 3 847 884). Therefore, reaching over 3 million in 2024, the number of prescriptions for injections exceeded the prepandemic level of 2.7 million in 2019. However, based on the prepandemic trends, it was still below the predicted count by half a million prescriptions per year.

**Table 1:** Trends in yearly prescription counts and rates for injections from 2015 to 2024

Year	Observed prescription counts	Observed year-on-year differences in counts (%)	Predicted prescription counts (95% CI)	Differences between observed and predicted counts (%)	Observed prescription rates	Observed year-on-year differences in rates (%)	Predicted prescription rates (95% CI)	Differences between observed and predicted rates (%)
2015	2 001 601	N/A	N/A	N/A	3504	N/A	N/A	N/A
2016	2 299 108	297 507 (15)	N/A	N/A	3986	482 (14)	N/A	N/A
2017	2 445 058	145 950 (6)	N/A	N/A	4181	195 (5)	N/A	N/A
2018	2 624 789	179 731 (7)	N/A	N/A	4434	253 (6)	N/A	N/A
2019	2 743 317	118 528 (5)	N/A	N/A	4579	145 (3)	N/A	N/A
2020	2 171 924	−571 393 (21)	2 977 956(2 905 348 to 3 050 565)	−806 031 (27)	3594	−985 (22)	4938(4811 to 5065)	−1344 (27)
2021	2 319 324	147 400 (7)	3 162 921(3 070 224 to 3 255 618)	−843 598 (27)	3805	211 (6)	5205(5043 to 5368)	−1400 (27)
2022	2 759 047	439 723 (19)	3 347 802(3 234 392 to 3 461 212)	−588 755 (18)	4466	660 (17)	5472(5273 to 5671)	−1007 (18)
2023	3 030 686	271 639 (10)	3 532 682(3 398 220 to 3 667 144)	−501 996 (14)	4842	376 (8)	5739(5504 to 5975)	−897 (16)
2024*	3 194 952	164 266 (5)	3 694 797(3 541 711 to 3 847 884)	−499 845 (13)	5047	205 (4)	5973(5705 to 6241)	−926 (16)

Rates are per 100 000 registered patients.

* The data for 2024 were available from January to September, therefore the yearly counts were calculated pro-rata for that year.

Correspondingly, we observed 424 963 (43%) more prescriptions for tablets in 2020 than in 2019 (table 2). The predicted difference for 2020 was 299 834 (27%), 1 415 315 observed versus 1 115 481 predicted (95% CI: 1 094 350 to 1 136 612). This trend continued in the following years, reaching over half a million more than expected prescriptions for tablets in 2024. The observed prescriptions count in 2024 was 2 230 729 versus 1 509 901 predicted (95% CI: 1 465 348 to 1 554 454). By 2024, there were over 2 million prescriptions issued for tablets, double the count of 2019.

**Table 2:** Trends in yearly prescription counts and rates for tablets from 2015 to 2024

Year	Observed prescription counts	Observed year-on-year differences in counts (%)	Predicted prescription counts (95% CI)	Differences between observed and predicted counts (%)	Observed prescription rates	Observed year-on-year differences in rates (%)	Predicted prescription rates (95% CI)	Differences between observed and predicted rates (%)
2015	593 056	N/A	N/A	N/A	1038	N/A	N/A	N/A
2016	720 216	127 160 (21)	N/A	N/A	1249	211 (20)	N/A	N/A
2017	824 604	104 388 (14)	N/A	N/A	1410	161 (13)	N/A	N/A
2018	921 819	97 215 (12)	N/A	N/A	1557	147 (10)	N/A	N/A
2019	990 352	68 535 (7)	N/A	N/A	1653	96 (6)	N/A	N/A
2020	1 415 315	424 963 (43)	1 115 481(1 094 350 to 1 136 612)	299 834 (27)	2342	689 (42)	1854(1817 to 1891)	488 (26)
2021	1 624 522	209 207 (15)	1 217 253(1 190 275 to 1 244 230)	407 269 (33)	2666	324 (14)	2012(1964 to 2060)	654 (33)
2022	1 788 786	164 264 (10)	1 318 977(1 285 971 to 1 351 983)	469 809 (36)	2895	230 (9)	2169(2111 to 2228)	726 (33)
2023	1 977 582	188 796 (11)	1 420 702(1 381 569 to 1 459 835)	556 880 (39)	3159	264 (9)	2327(2257 to 2396)	832 (36)
2024*	2 230 729	253 147 (13)	1 509 901(1 465 348 to 1 554 454)	720 828 (48)	3524	365 (12)	2465(2386 to 2544)	1059 (43)

Rates are per 100 000 registered patients.

* The data for 2024 were available from January to September, therefore the yearly counts were calculated pro-rata for that year.

The changes in prescription counts were reflected in rates. The initial drop in 2020 in prescriptions for injections, by nearly 1000 prescriptions per 100 000 patients, levelled up by 2024 to the prepandemic rates. The observed rate in 2024 was 5047, exceeding the 2019 rate of 4579, but representing a predicted deficit of 926 (16%) prescriptions per 100 000 patients as estimated from the predicted rate of 5973 (95% CI: 5705 to 6241). In contrast, for tablets, in 2020, there were 488 (26%) more prescriptions per 100 000 than expected. This increased in 2024 to 1059 (43%) more prescriptions than expected. The observed rate in 2024 was 3524 versus 2465 predicted (95% CI: 2386 to 2544). The observed rate for tablets doubled from 1653 in 2019 to 3524 prescriptions per 100 000 people in 2024.

Despite the drop in prescriptions for injections and the increase in prescriptions for tablets, there were more prescriptions for injections issued than tablets every year across the study period. In 2023 and 2024 alike, there were 3 million prescriptions for injections and 2 million prescriptions for tablets. However, the proportion shifted over time towards tablets. In 2019, there were three times more prescriptions for injections than tablets, nearly 3 million (at a rate of over 4579 prescriptions per 100 000 patients) versus 1 million (rate 1653). By 2024, this proportion decreased to one and a half more prescriptions for injections than tablets. In 2024, there were over 3 million prescriptions for injections and over 2 million for tablets.

Figure 1 shows that the average quantity of ampoules was steadily decreasing across the study period from around 1.6 to 1.4 ampoules per prescription. This was similar for tablets, the average quantity of tablets per prescription decreased from 55 in 2015 to 48 in 2024. The trend for injections was not affected by the pandemic. However, we observed a temporary increase in the number of tablets prescribed in 2020, with a peak in March 2020 at 58 tablets per prescription.

### Regional trends

**Figure 2:**
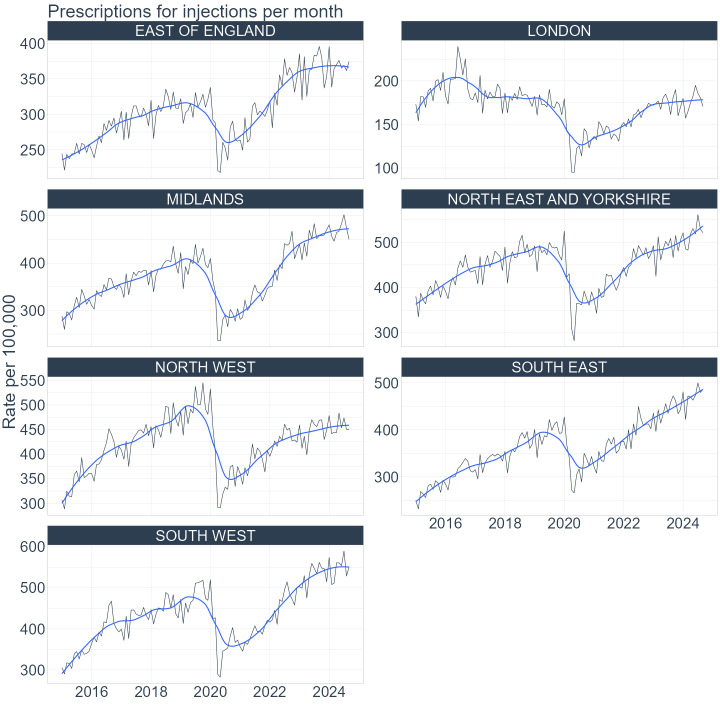
Regional trends in prescribing of injections from January 2015 to September 2024. Monthly prescription rates per 100 000 registered patients are presented for regions in England: East of England, London, Midlands, North East and Yorkshire, North West, South East and South West. The black line represents monthly rates and the blue line represents 3-point moving average smoothed trends.

**Figure 3:**
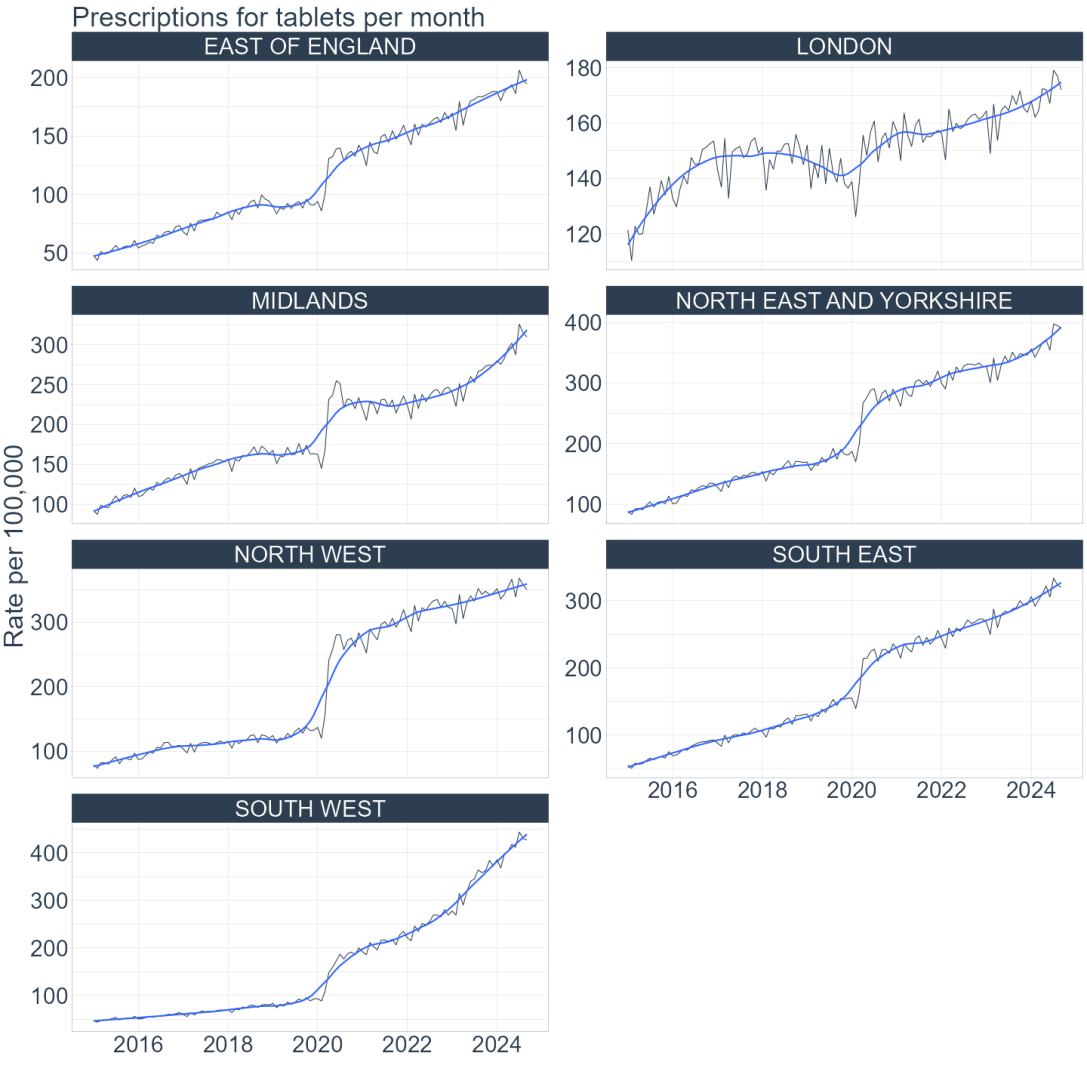
Regional trends in prescribing of tablets from January 2015 to September 2024. Monthly prescription rates per 100 000 registered patients are presented for regions in England: East of England, London, Midlands, North East and Yorkshire, North West, South East and South West. The black line represents monthly rates and the blue line represents 3-point moving average smoothed trends.

The regional trends resembled the national trends. The sudden drop in prescriptions for injections as well as the sudden increase in prescriptions for tablets was observed in 2020 for all regions in England. The trends in monthly rates per 100 000 for injections are presented in figure 2 and for tablets in figure 3. The raw monthly counts of prescriptions for injections are presented in the online supplemental figure S1 and for tablets in the online supplemental figure S2. The steady upward trend in prescriptions for injections, after the initial drop in 2020, was observed in all the regions. However, some regions, for example, the North West, did not recover to the prepandemic levels as quickly as the other regions.

The rates of prescriptions varied between the regions. At the end of the study period, London had the lowest number of prescriptions issued for both forms. In London, there were around 19 000 prescriptions issued per month for injections at the rate of around 180 per 100 000 patients and 18 000 prescriptions for tablets at the rate of 170 per 100 000 patients. In comparison, in the South West, which was the region with the highest number of prescriptions, there were nearly 35 000 prescriptions for injections per month at the rate of 550 per 100 000 and around 22 000 prescriptions for tablets and the rate was 360 per 100 000 patients.

## Discussion

### Summary

We observed a sharp dip in prescriptions for injections of vitamin B_12_ at the beginning of 2020 and a simultaneous rapid increase in tablet usage nationally and across all regions in England. These changes coincided with the outbreak of the COVID-19 pandemic. From 2020 onwards, the prescriptions for both injections and tablets were increasing steadily with injections returning to the pre-2020 levels by the end of the study period and prescriptions for tablets doubling between 2019 and 2024. Despite the changes, there were more prescriptions for injections than tablets issued across the study period. However, the proportion shifted from three times more prescriptions for injections than tablets in 2019 (3 million vs 1 million) to one and a half times more prescriptions for injections than tablets in 2024 (3 million vs 2 million).

### Strengths and limitations

This is the first national study documenting patterns in the prescribing of vitamin B_12_, focusing on a comparison between tablets and injections before and after the pandemic. An important strength of this study was in the size and completeness of the data set. We accessed prescribing data from all GP practices in England achieving 100% national coverage. Data were nearly real-time, so this study presented current trends. We extracted the data in December 2024 showing trends for up to September 2024. We used a transparent and open research approach. Data used in this study were open access and freely available for reproduction and scrutiny of our analyses and findings. The original data are published monthly by the NHS and this facilitates future updates to monitor further changes in prescribing. With the release of the new NICE guidelines focusing on the diagnosis and management of vitamin B_12_ deficiency (published in March 2024)^15^ and as we continue learning from the pandemic, it becomes critical to replicate this study in the future.

We also note some limitations. This data set was based in England only. Similar studies from other countries are needed to understand the global perspective. Data were anonymised and only aggregate prescription counts were available. Therefore, the information on patient demographics and disorders treated with B_12_ supplements was unknown and could not be considered. Furthermore, we could not access information on B_12_ supplements purchased by patients online or over the counter. This is a potentially growing trend and could be a source of bias leading to underestimated values for tablets and injections.^23^ Future studies are needed to evaluate this trend. The information on doses prescribed was not available which makes comparison of quantities challenging. We considered all the tablet formulations, including all strengths. Future research investigating switches between strengths of tablets could be of value. Although we used practice list sizes to calculate rates and enable comparisons with other published studies, our approach was limited because the same-size populations may differ in the characteristics of patients. A methodology for adjusting for age and sex in populations exists but this is imperfect and resource-intensive, so not available for this study.^24^

### Results in the context of prior work

We used a data set with 100% national coverage in England to show the changes in prescribing patterns from 2015 to 2024. We accessed the number of prescriptions and number of units (tablets and ampoules) per prescription. We showed a shift from vitamin B_12_ injections towards tablets. More research is needed to evaluate the effect of these changes on symptoms and quality of life of patients, including fatigue, peripheral neuropathy, pain, impaired sleep, cognition and mental health.^25^ A survey in the UK, by Seage and Semedo, investigated the impact on people with pernicious anaemia (n=329) who experienced a suspension of injections, with tablets being offered as an alternative.^26^ They reported uncertainty about the long-term effects on health and well-being and dissatisfaction with healthcare. The study reported a need for more support in the management of pernicious anaemia beyond the pandemic.

The Royal College of General Practitioners advised during the COVID-19 pandemic that selected patients should be taught to self-administer B_12_ injections.^18^ However, the British Society for Haematology recommended against patients switching to self-administration during the pandemic due to limited opportunities for support and supervision.^17^ A UK survey (n=1297) by Tyler *et al* found that 40% of people with B_12_ deficiency, including pernicious anaemia, self-medicated via injection (this was defined as buying a supply online without a prescription).^23^ People reported stigma, let down and the lack of a patient-centred approach in primary care. This eroding trust promoted the need to self-medicate to regain quality of life and negatively affected the perception of healthcare. Most importantly, postponing injections for people on maintenance treatment could expose patients to worsening symptoms and in extreme cases irreversible neurological damage that could potentially be avoided by support and self-administration.^27 28^

Injections remained the most popular option throughout the study period. There were 3 million prescriptions for injections issued in 2019 and in 2024. In comparison, there were 2 million prescriptions for tablets issued in 2024, the most since 2015 and double the amount in 2019. We also showed that on average there were around 1.4 ampoules and 56 tablets per prescription. This is relevant, because for the long-term management of vitamin B_12_ deficiency, one ampoule is needed every 2–3 months^19^ and 56 tablets usually provide 1–2 months of treatment.^20^ At 1.4 ampoules per prescription, we expect approximately three to four prescriptions per year and at 56 tablets, we expect 6–12 prescriptions per year. Therefore, although we did not have the exact dosage information, we concluded that for oral to be the major route we should expect the number of prescriptions to be twice that of the injections.

### Implications for future research and practice

Inadequately treated B_12_ deficiency can lead to serious physical and psychological complications. Traditionally, injections have been considered the best option unless there is evidence of dietary deficit. Many of the B_12_ deficiency conditions, for example, pernicious anaemia, are chronic and require lifelong supplementation. The administration of injections requires more resources than dispensing tablets. Even before the pandemic, there had already been an ongoing discussion about the limited evidence supporting the benefit of injections versus tablets for B_12_ deficiency.^12^,^1525^ This included inconclusive evidence comparing the safety, tolerability, clinical effectiveness and cost effectiveness of both types of treatment. The COVID-19 pandemic brought a rapid review in prescribing guidelines^17 18^ and as we show in this study, greatly accelerated the shift from injections to tablets. However, the pandemic-related changes focused on reducing primary care appointments and protecting patients from COVID-19 infections. Therefore, the challenges associated with the limited evidence in relation to patient outcomes remain pertinent. The COVID-19 pandemic should also accelerate the important opportunities for more research in this area.

## Conclusions

For many years, primary care in the UK has been severely affected by staff shortages and the COVID-19 pandemic exacerbated this crisis. In this study, we show how the COVID-19-related restrictions drove changes to standards of care and accelerated the switch from injections to tablets for many patients with vitamin B_12_ deficiency. We show a rapid shift in 2020. We also show a continuous increase in prescriptions for both injections and tablets. Injections were back to the prepandemic levels by 2024. This is the first national study documenting those patterns. The pandemic-related changes were dictated by the availability of resources so more evidence on clinical benefits and harms is required. A NICE guideline on the diagnosis and management of B_12_ deficiency was published in March 2024.^15^ More evidence comparing the safety, tolerability and clinical and cost-effectiveness of injections and tablets in different B_12_ disorders is needed to establish best practices.

## supplementary material

10.1136/bmjopen-2024-093748 online supplemental file 1

## Data Availability

Data are available in a public, open access repository.
