# Supplementary material for: Vitamin B12 prescribing from 2015 to 2024 in English general practice: an observational study to investigate the switch from injections to tablets
Source: BMJ Open. 2025 Feb 12;15(2):e093748. doi: 10.1136/bmjopen-2024-093748 (PMC11822439; doi:10.1136/bmjopen-2024-093748)

Supplemental material

Supplemental Table S1. The list of tablet vitamin B12 formulations. British National Formulary (BNF) formulation names and codes.

BNF name	BNF code
Acyanocomin 500microgram tablets	0901020D0BMAAAK
Behepan 1mg tablets	0901020D0BDAAAF
CyanocoMinn 1mg modified-release tablets	0901020D0BIAAAH
Orobalin 1mg tablets	0901020D0BLAAAF
CyanocoB12 1mg tablets	0901020D0BKABAF
Solgar Vitamin B12 1000microgram sublingual nuggets	0901020D0BGAAAI
Cyanocobalamin 1mg modified-release tablets	0901020D0AAAHAH
Cyanocobalamin 100microgram tablets	0901020D0AAAJAJ
HealthAid Vitamin B12 1000microgram modified-release tablets	0901020D0BEAAAH
SunVit-Pro Vitamin B12 50microgram tablets	0901020D0BPAAAE
CyanocoMinn 100microgram tablets	0901020D0BIADAJ
Cyanocobalamin 1mg sublingual tablets sugar free	0901020D0AAAIAI
CyanocoB12 1mg modified-release tablets	0901020D0BKAAAH
CyanocoB12 50microgram tablets	0901020D0BKACAE
Lamberts Vitamin B12 1000microgram tablets	0901020D0BFAAAF
Cyanocobalamin 50microgram tablets	0901020D0AAAEAE
CyanocoB12 500microgram tablets	0901020D0BKAEAK
CyanocoMinn 50microgram tablets	0901020D0BIABAE
CyanocoMinn 1mg tablets	0901020D0BIACAF
Lamberts Vitamin B12 100microgram tablets	0901020D0BFABAJ
Cyanovit-B12 1000microgram tablets	0901020D0BHAAAF
CyanocoB12 100microgram tablets	0901020D0BKADAJ
Cyanocobalamin 1mg tablets	0901020D0AAAFAF
Cyanocobalamin 500microgram tablets	0901020D0AAAKAK
Cytacon 50microgram tablets	0901020D0BCAAAE
Quest Vitamin B12 High Potency 1000microgram tablets	0901020D0BJAAAF

Supplemental Table S2. The list of injectable vitamin B12 formulations. British National Formulary (BNF) formulation names and codes.

BNF name	BNF code
Neo-Cytamen 1000micrograms/1ml inj ampoules	0901020N0BBAAAB
Cobalin-H 1mg/1ml solution for injection ampoules	0901020N0BDAAAB
Hydroxocobalamin 1mg/1ml solution for injection ampoules	0901020N0AAABAB
Cyanocobalamin 1mg/1ml solution for injection ampoules	0901020D0AAADAD
Cytamen 1000micrograms/1ml solution for injection ampoules	0901020D0BBABAD

Supplemental Figure S1. Regional trends in prescribing of injections from January 2015 to September 2024. Monthly prescription counts are presented for regions in England: East of England, London, Midlands, North East and Yorkshire, North West, South East, and South West. The black line represents raw monthly counts, and the blue line represents 3-point moving average smoothed trends.

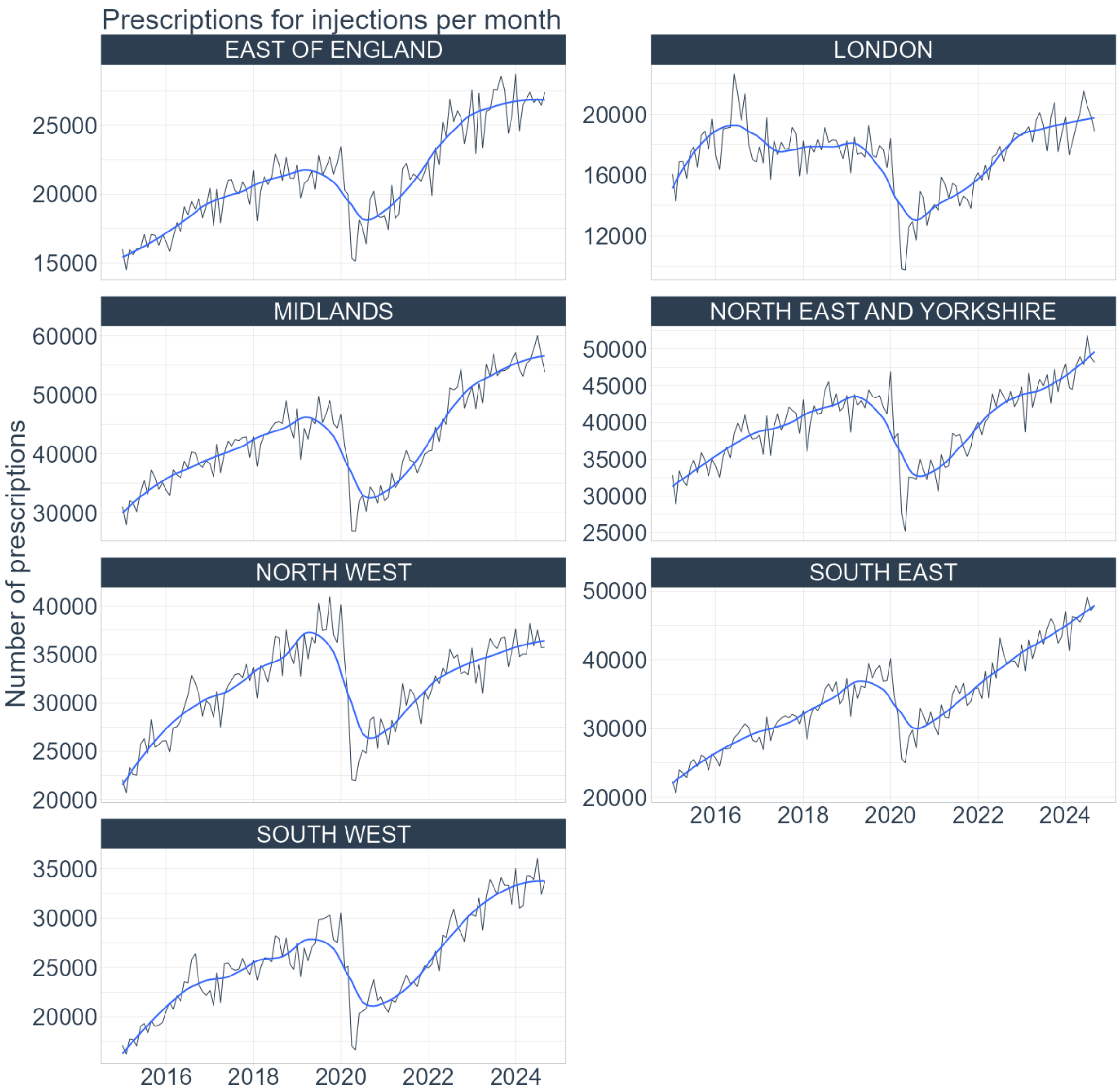

Supplemental Figure S2. Regional trends in prescribing of tablets from January 2015 to September 2024. Monthly prescription counts are presented for regions in England: East of England, London, Midlands, North East and Yorkshire, North West, South East, and South West. The black line represents raw monthly counts, and the blue line represents 3-point moving average smoothed trends.

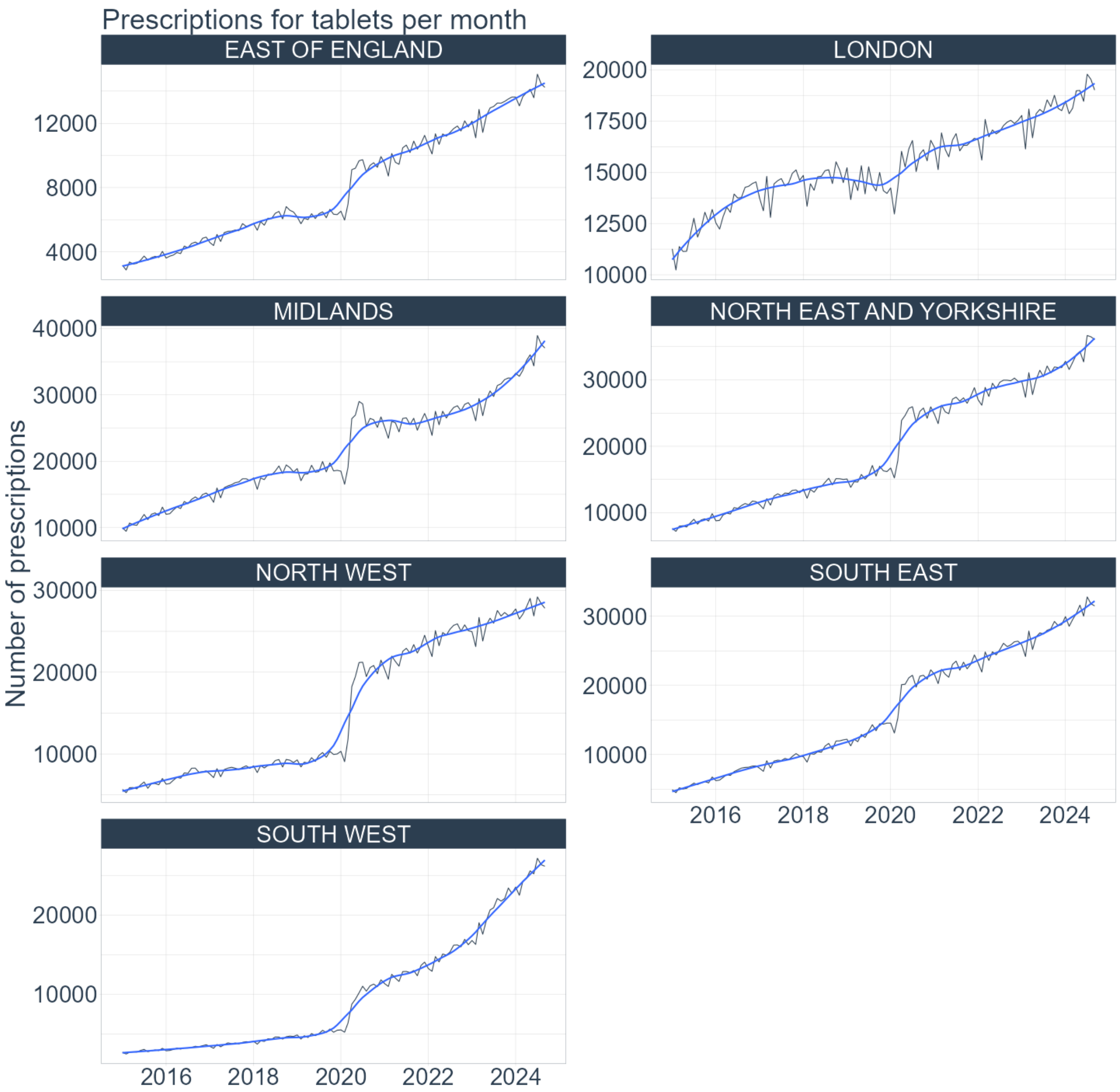

Supplement: online supplemental file 1 [file bmjopen-15-2-s001.pdf]
