# Supplementary material for: Vitamin B12 prescribing from 2015 to 2024 in English general practice: an observational study to investigate the switch from injections to tablets
Source: BMJ Open. 2025 Feb 12;15(2):e093748. doi: 10.1136/bmjopen-2024-093748 (PMC11822439; doi:10.1136/bmjopen-2024-093748)
Supplement: Supplementary file 2 [file bmjopen-2024-093748.reviewer_comments.pdf]

PEER REVIEW HISTORY

BMJ Open publishes all reviews undertaken for accepted manuscripts. Reviewers are asked to complete a checklist review form and are provided with free text boxes to elaborate on their assessment. These free text comments are reproduced below.

ARTICLE DETAILS

Title (Provisional)

Vitamin B12 prescribing from 2015 to 2024 in English general practice: an observational study to investigate the switch from injections to tablets

Authors

Tank, Nikita; Wood, Christopher; Ahankari, Anand; Mackenna, Brian; Walker, Alex; Lemanska, Agnieszka

---

VERSION 1 - REVIEW

**Reviewer**	**1**
**Name**	**Selinger, Eliška**
**Affiliation**	**Centre for Research on Diabetes, Metabolism and Nutrition of Third Faculty of Medicine, Charles University, Dept. of Medicine 2, University Hospital Kralovske Vinohrady, Prague 10000, Czech Republic**
**Date**	**20-Oct-2024**
**COI**	**None**

Thanks for submitting this clear and focused analysis of the prescription trends in vitamin B12. The overall aim of the analysis is clear and results supports the conclusion. I have only minor comments/questions related to some parts of the manuscript or the overall background/discussion of its results.

- Why only two forms (hydroxocobalamin and cyanocobalamin) were selected? Is it because they are the most prevalent/only available for prescription? (In my region there are more chemical forms on the market)

- As the data for 2024 are not fully available, maybe it would be better to just end the analysis in 2023? I am not sure if it is justifiable to calculate the

---

**Reviewer**	**2**
**Name**	**Ward, Nicola**
**Affiliation**	**De Montfort University, Leicester School of Pharmacy**
**Date**	**07-Nov-2024**

COI None

An interesting paper, which usefully explores trends in the prescribing of B12 supplementation over a significant time period. The authors acknowledge some important limitations in their interpretation of the data. However, there are some further limitations relating to operational aspects of prescribing and administration of supplementation that need consideration for the discussion and conclusions to be recognised as applicable and relevant to current practise.

p.3 abstract background: It is unclear what the evidence is for the statement "the improving understanding of the underlying causes of B12 deficiency....." resulted in a shift from injections to tablets. This is not evidenced in this study. However, it is acknowledged that some GP practices and ICBs have been promoting a move to oral administration for a number of years, regardless of the cause of the B12 deficiency.

p3.38 abstract results: typo noted- "tables" rather than tablets

p4.14 pernicious anaemia manifests with megaloblastic anaemia- should be noted that not all cases present with macrocytosis, and this misconception can result in missed diagnoses.

p4.34-36 good to note that the authors acknowledge the limited quantity and quality of evidence available. It would be useful to cite some of the papers referred to in this paragraph.

p4.53-54 statement referred to here regarding storage of B12 in the liver was later revised by the BCSH as it resulted in many patients having treatment stopped long-term.

Limitations:

The data relating to prescriptions of injections does not appear to consider the different operational models for prescribing and administering B12 injections in primary care. Some prescribers will write prescriptions for individual patients, which will be dispensed in the local pharmacy and then taken by the patient to their appointment at the surgery for administration or self-administered at home. This may be prescribed as an original pack of 5 ampoules or as individual ampoules to avoid accidental loss in between appointments. In many cases the patient does not have to visit their pharmacy for a prescription to be dispensed, but attends the surgery for their injection and receives an injection via a Patient Group Direction (PGD) or Patient Specific Direction (PSD). It is unclear how these different models of care would influence the interpretation of the prescription data as reported in this study.

Appendix 1 lists a range of different oral cobalamin products of varied strengths. The data in this study is based on a presumption that patients are taking one tablet per day. However, patients prescribed lower strength products may be told to take more than one tablet a day,

as many of the higher strength products are not available on local formularies due to them not being licensed medicines, but nutritional supplements.

In addition, many patients are requested to purchase their own oral supplements to avoid the cost of prescribing. This would be very challenging to quantify , but may contribute towards the difference between the level of prescribing of oral supplements vs. what the authors expected to see.

p15.16 ambiguous- this paper was specifically about patient experiences of treatment during the pandemic rather than healthcare in general.

p15.48 one ampoule every 3 months- BNF states every 2-3 months, with some patients requiring more frequent treatment, which some GPs will prescribe for them.

p15.49 56 tablets- presumption that patient is taking one tablet per day.

VERSION 1 - AUTHOR RESPONSE

Reviewer: 1 Comments to the Author:	
Thanks for submitting this clear and focused analysis of the prescription trends in vitamin B12. The overall aim of the analysis is clear and results supports the conclusion. I have only minor comments/questions related to some parts of the manuscript or the overall background/discussion of its results.	Thank you for taking the time to provide the review.
- Why only two forms (hydroxocobalamin and cyanocobalamin) were selected? Is it because they are the most prevalent/only available for prescription? (In my region there are more chemical forms on the market)	Thank you for this question. We amended the text and it now says:<br>“We included the two main forms of vitamin B12, hydroxocobalamin (British National Formulary (BNF) code 0901020N0) [19], and cyanocobalamin (BNF code 0901020D0) [20]. Only those two forms were available via prescriptions from the NHS.”<br><br>Hydroxocobalamin and cyanocobalamin are the only B12 forms available on prescriptions in the UK. Some other forms are available, such as methylcobalamin or adenosylcobalamin, but they are only available in dietary supplements purchased online and over the counter. We could not capture this trend.<br><br>We also added the following sentence in the limitations section:<br>“Furthermore, we could not access information on B12 supplements purchased by patients online or

	over the counter. This is a potentially growing trend and could be a source of bias leading to underestimated values for tablets and injections [23]. Future studies are needed to evaluate this trend.”
- As the data for 2024 are not fully available, maybe it would be better to just end the analysis in 2023? I am not sure if it is justifiable to calculate the	Thank you for this comment. We retained 2024 in the study and provide the rationale below.<br><br>We originally presented data up to May 2024, and now, within this revision, we have data available up to September 2024. We refreshed the graphs and the information in the text.<br><br>On the graphs, we present monthly rates, so the cutoff date does not have to be the end of one year, and because we investigate changes in trends over time, it was important to present the most recent data, as fresh as possible, and so we retained 2024.<br><br>We understand that the main concern from the reviewer refers to us calculating prescription counts and rates for 2024, pro rata, based on the available data. We estimated the values for the whole 2024 because it was necessary to make it comparable with the other years, and this approach provides relevant and valid information about the trends in 2024. Since we now have nine months of data (Jan to September) this presents a strong case to retain 2024 and to show the most up to date trends. It is important to note that we do not predict, forecast or project into the future, we estimate the values for 2024 as it would be in the first 9 months. This is a conservative approach and presents true values (just extrapolated to the whole year) and has been used before in similar studies. We hope that this explanation satisfies this point.
Reviewer: 2 Comments to the Author:	
An interesting paper, which usefully explores trends in the prescribing of B12 supplementation over a significant time period. The authors acknowledge some important limitations in their interpretation of the data. However, there are some further limitations relating to operational aspects of prescribing and administration of supplementation that need consideration for the discussion and conclusions to be recognised as	Thank you for taking the time to read the manuscript and to provide feedback.

applicable and relevant to current practise.	
p.3 abstract background: It is unclear what the evidence is for the statement "the improving understanding of the underlying causes of B12 deficiency….." resulted in a shift from injections to tablets. This is not evidenced in this study. However, it is acknowledged that some GP practices and ICBs have been promoting a move to oral administration for a number of years, regardless of the cause of the B12 deficiency.	Thank you for this context. We rephased the paragraph as follows:<br>"Traditionally, intramuscular vitamin B12 injections were considered by patients and clinicians the most effective treatment option for B12 deficiency. The improving understanding of the condition paired with the restricted National Health Service (NHS) resources, resulted in a shift from injections towards tablets. The COVID-19 pandemic accelerated this change, while healthcare services were adapted to reduce COVID-19 transmission. This included new guidelines on vitamin B12 prescribing where injections were substituted by tablets."<br><br>We note that our original statement was unclear as it meant to convey that even before the pandemic there was a move towards injections from tables, and that the pandemic accelerated this shift, especially in dietary deficiency group. Guidelines were more unclear/vague (especially at the start of the pandemic) for people with deficiency related to impaired absorption.
p3.38 abstract results: typo noted- "tables" rather than tablets	Thank you, this has now been corrected.
p4.14 pernicious anaemia manifests with megaloblastic anaemia- should be noted that not all cases present with macrocytosis, and this misconception can result in missed diagnoses.	Thank you, amended. Changed from:<br>This includes pernicious anaemia, a rare condition that manifest with megaloblastic (abnormally large and immature) red blood cells [6-8].<br><br>The paragraph now states:<br>"In most people the condition [the deficiency] is hidden and difficult to diagnose but affecting quality of life. In chronic and severe cases, B12 deficiency can lead to serious and potentially life-threatening disorders including neurological damage and haematological abnormalities. This includes neuropathy, ataxia and megaloblastic anaemia (abnormally large and immature red blood cells) [6-8]. Pernicious anaemia is a rare but irreversible condition caused by malabsorption which can be linked to gastrointestinal autoimmune disorders or surgical interventions. It requires lifelong B12 supplementation…"
p4.34-36 good to note that the authors acknowledge the limited quantity and quality of evidence available. It would	Amended. References were added.

be useful to cite some of the papers referred to in this paragraph.	
p4.53-54 statement referred to here regarding storage of B12 in the liver was later revised by the BCSH as it resulted in many patients having treatment stopped long-term.	Thank you for this information. We agree and recognise that it was important not to repopulate these statements and therefore, we removed the following from the manuscript:<br>"These [interim] guidelines assured healthcare professionals and patients that B12 was stored in the liver, and that the injections could be postponed. They specified that oral supplementation for dietary deficiency was as effective as injections, and that for non-dietary cause oral formulations at a high dose could be given as an interim measure before injections can be restored."<br><br>We wish to note that we followed the updates from https://pernicious-anaemia-society.org/pernicious-anaemia/bsh-guidance-on-b12-changed/, and that for dietary deficiency, the revised BSH guidelines (dated: 24.04.2020) still state the following: "However, many of these patients may be vitamin B12 replete with adequate levels within the liver, and therefore may be able to safely stop taking vitamin B12 supplements possibly for up to a year" https://b-s-h.org.uk/media/18259/bsh-guidance-b12-replacement-covid-1924042020finalversion2020-4-3.pdf
Limitations:<br>The data relating to prescriptions of injections does not appear to consider the different operational models for prescribing and administering B12 injections in primary care. Some prescribers will write prescriptions for individual patients, which will be dispensed in the local pharmacy and then taken by the patient to their appointment at the surgery for administration or self-administered at home. This may be prescribed as an original pack of 5 ampoules or as individual ampoules to avoid accidental loss in between appointments. In many cases the patient does not have to visit their pharmacy for a prescription to be dispensed, but attends the surgery for their injection and receives an injection	Thank you for this comment. We note that the data from both models of prescribing/administration (including FP10s, PGDs and PSDs) are captured in the dataset. More information can be found here https://openprescribing.net/faq/#fp34.

via a Patient Group Direction (PGD) or Patient Specific Direction (PSD). It is unclear how these different models of care would influence the interpretation of the prescription data as reported in this study.	
Appendix 1 lists a range of different oral cobalamin products of varied strengths. The data in this study is based on a presumption that patients are taking one tablet per day. However, patients prescribed lower strength products may be told to take more than one tablet a day, as many of the higher strength products are not available on local formularies due to them not being licensed medicines, but nutritional supplements.	Thank you for this comment.<br>Amended from:<br>“56 tablets usually provide two months treatment.<br>To:<br>“56 tablets usually provide one to two months treatment.”<br><br>We note that we included all the available tablet formulations and they are listed in appendix 1. However, the vast majority of prescribed products were the cyanocobalamin 50 and 100 microgram tablets.<br><br>We also note that the main presumptions of the study were not based around the quantities, they were based on a direct comparison of the crude prescription numbers and rates per 100,000. For example, in the conclusions we state:<br>“Despite the changes, there were more prescriptions for injections than tablets issued across the study period. However, the proportion shifted from three times more prescriptions for injections than tablets in 2019 (3 million versus 1 million), to one and a half times more prescriptions for injections than tablets in 2024 (3 million versus 2 million).”<br><br>Only one paragraph in the manuscript is based around the presumption of quantities. This paragraph is pasted in the last row of this table. We edited it in accordance with the feedback and we think that it adds value in the discussion. However, we also agree that it is relatively speculative and if the reviewers suggest, we are happy to remove this paragraph from the manuscript.
In addition, many patients are requested to purchase their own oral supplements to avoid the cost of prescribing. This would be very challenging to quantify , but may contribute towards the difference between the level of prescribing of oral supplements vs. what the authors expected to see.	Thank you for this comment. Some patients were/are requested to purchase, and some also self-medicate and purchase either tables or injections (methylcobalamin) and self-inject. We were not able to capture these trends.<br><br>We added the flowing statement to the limitations section:

	"Furthermore, we could not access information on B12 supplements purchased by patients online or over the counter. This is a potentially growing trend and could be a source of bias leading to underestimated values for tablets and injections [23]. Future studies are needed to evaluate this trend."
p15.16 ambiguous- this paper was specifically about patient experiences of treatment during the pandemic rather than healthcare in general.	Amended.<br>Revised from:<br>"A survey in the UK, by Seage and Semedo (2021), investigated the impact on patients' experience of healthcare. People with pernicious anaemia (n = 329) experienced a suspension of injections, with tablets being offered as an alternative [25]."<br>To:<br>"A survey in the UK, by Seage and Semedo (2021), investigated the impact on people with pernicious anaemia (n = 329) who experienced a suspension of injections, with tablets being offered as an alternative [25]."
p15.48 one ampoule every 3 months- BNF states every 2-3 months, with some patients requiring more frequent treatment, which some GPs will prescribe for them.	Amended from:<br>"This is relevant, because for the long-term management of vitamin B12 deficiency, one ampule is needed every three months [19], and 56 tablets usually provide two months treatment [20]."<br>To:<br>"This is relevant, because for the long-term management of vitamin B12 deficiency, one ampule is needed every two to three months [19], and 56 tablets usually provide one to two months treatment [20]."
p15.49 56 tablets- presumption that patient is taking one tablet per day.	Thank you for this comment. We amended the whole statement and it now states as follows:<br>"We also showed that on average there were around 1.4 ampoules and 56 tablets per prescription. This is relevant, because for the long-term management of vitamin B12 deficiency, one ampule is needed every two to three months [19], and 56 tablets usually provide one to two months treatment [20]. At 1.4 ampules per prescription, we expect approximately three to four prescriptions per year, and at 56 tablets we expect six to twelve prescriptions per year. Therefore, although we did not have the exact dosage information, we concluded that for oral to be the major route we should expect the number of prescriptions to be twice that of the injections.<br><br>The limitations section also contains the following information:

	“The information on doses prescribed was not available which makes comparison of quantities challenging. We considered all the tablet formulations, including all strengths.”
